# Healthcare social network research and the ECHO model™: Exploring a community of practice to support cultural brokers and transfer cultural knowledge

**DOI:** 10.1186/s12913-024-11024-w

**Published:** 2024-05-01

**Authors:** Phil Nixon, Chiara Broccatelli, Perrin Moss, Sarah Baggio, Angela Young, Dana Newcomb

**Affiliations:** 1 Present Address: Integrated Care, Children’s Health Queensland Hospital and Health Service, 501 Stanley Street, South Brisbane, QLD 4101 Australia; 2 Present Address: Department of Social and Cultural Anthropology, Universitat Autònoma de Barcelona, Barcelona, 08193 Spain; 3 Institute for Social Science Research, The University of Queensland, Indooroopilly, QLD 4068 Australia; 4 School of Health and Rehabilitation Sciences, The University of Queensland, Saint Lucia, QLD 4072 Australia; 5 General Practice Clinical Unit, The University of Queensland, Herston, QLD 4029 Australia

**Keywords:** Project ECHO, Community of Practice, Aboriginal and Torres Strait Islander, Aboriginal and Torres Strait Islander Health Worker, Social network research, Integrated Care, Virtual learning, Workforce development, Cultural broker

## Abstract

**Background:**

Project ECHO^®^ networks at Children’s Health Queensland Hospital and Health Service (CHQHHS) are communities of practice designed to mitigate services and systems fragmentation by building collaborative partnerships addressing priority child and youth health needs. Aboriginal and Torres Strait Islander people experience the negative impacts of fragmentation in addition to historical challenges of absent or culturally inappropriate health services. Access to culturally safe and responsive services can be improved by engaging Aboriginal and Torres Strait Islander Health Workers and similar roles in an online community of practice, supporting the integration of cultural and clinical knowledge and self-determination of Aboriginal and Torres Strait Islander consumers in decisions affecting their health.

Analysing professional support networks and knowledge sharing patterns helps identify enablers and barriers to partnerships. Using social network research, the multilevel network inclusive of ECHO network members and their colleagues was studied to identify interdisciplinary and cross-sector advice exchange patterns, explore the position of cultural brokers and identify common relational tendencies.

**Methods:**

Social network theories and methods informed the collection of network data and analysis of advice-seeking relationships among ECHO network members and their nominees. Registered members from two ECHO networks were invited to complete the Qualtrics survey. Networks analysed comprised 398 professionals from mainstream health, Aboriginal and Torres Strait Islander Community Controlled Health Organisation, education, disability and child safety service settings.

**Results:**

Brokers were well represented, both those who hold knowledge brokerage positions as well as cultural brokers who incorporate clinical and cultural knowledge enabling holistic care for Aboriginal and Torres Strait Islander patients (38 individuals, 17% of network). Professionals who occupy brokerage positions outside the ECHO network tend to be more connected with co-members within the network.

**Conclusions:**

This study is the first application of contemporary social network theories and methods to investigate an ECHO network. The findings highlight the connectivity afforded by brokers, enabling the coordination and collaboration necessary for effective care integration. Inclusion of cultural brokers in an ECHO network provides sustained peer group support while also cultivating relationships that facilitate the integration of cultural and clinical knowledge.

**Supplementary Information:**

The online version contains supplementary material available at 10.1186/s12913-024-11024-w.

## Background

The ECHO model™ was developed at the University of New Mexico to address inequities in access to specialty care [1, 2]. In each ECHO network, regular multipoint videoconference sessions combine de-identified case discussions with brief presentations. Problem-based case discussions elicit valuable information from the perspectives of ECHO participants and panel members, to build collective knowledge of clinical conditions, system and sector navigation, and culturally appropriate service provision, as well as to facilitate connections and enhanced coordination between providers. The model is inherently learner-centric, as panels are attentive to emergent participant learning needs and design future content to address group priorities. To provide clarity on terminology used in relation to the ECHO model, social network research and Aboriginal and Torres Strait Islander healthcare, please refer to Table 1.

**Table 1:** Key terms by subject area

**ECHO Community of Practice descriptive term**	
**Community of Practice (CoP)**	A group with a shared interest in a real-life problem, who choose to interact regularly to learn from and with each other [3]
**ECHO Network**	A virtual CoP hosted using the ECHO model™. In this manuscript, ECHO network and ECHO CoP are used interchangeably
**Participant**	A member of the ECHO CoP with an interest in the focus area of the ECHO CoP
**Panel Member**	An appointed member of the ECHO CoP who provides dedicated support and mentorship. Generally considered an expert in the focus area of the ECHO CoP
**ECHO CoP Member**	Any individual ECHO Participant or Panel Member, as defined above
**Social network research term**	
**Broker/ brokerage**^a^	An individual who connects otherwise unconnected individuals in the network, enabling flow of advice, knowledge or other resource
**Cluster**	Group of individuals with a higher density of relationships
**Network**	Relationships occurring in a given context
**Node**	An individual in the network
**Respondent**^c^	Registered ECHO Participant or Panel Member who completed the network survey
**Nominee**^c^	Individual who was nominated by a respondent during the network survey
**Tie**	Relationship between two individuals
**Aboriginal and Torres Strait Islander healthcare term**	
**Cultural broker/ cultural brokerage**^b^	An individual who can facilitate the interaction between a person or group of people from one culture to another [4]

Note concept overlap, with clarification of distinct meanings provided by subject area. The distinction between brokerage and cultural brokerage is important to an understanding of this paper

^a^Social network research methods are used in this work to identify and analyse brokers within the ECHO network

^b^Described as “part-interpretative, part-advocacy” [5], the role of a cultural broker in a healthcare context is nuanced. A cultural broker is an Aboriginal and/or Torres Strait Islander person who has an understanding of the world views, cultural values, beliefs and practices of different cultural groups [4] and draws on this knowledge to foster healthcare team provision of holistic care. For instance, in a policy setting a cultural broker uses their lived experience and cultural knowledge to provide advice about how their cultural group will be impacted by or perceive the implementation of that policy

^c^Care has been taken with terminology referring to the study group throughout the manuscript. The terms respondent and nominee are appropriate depending on the type and level of analysis

ECHO networks addressing a multitude of health focus areas have demonstrated positive impacts on health care provider knowledge, self-efficacy, and professional isolation [6–10]. A small number of studies have demonstrated changes in care practice and patient-level health outcomes [11]. In addition to the need for further research on patient and community health outcomes, there is a need to understand knowledge translation patterns, including how advice is sought, gained, and applied to patient care. Anecdotal evidence from ECHO participants and panel members at Children’s Health Queensland Hospital and Health Service (CHQHHS) indicates new and strengthened socio-professional connections between providers from diverse geographical regions, services and sectors. Positive outcomes based on these connections have included new organisational partnerships to progress shared priorities, primary and tertiary team collaboration to streamline both continuity and coordination of care, and the adoption of new models of care in regional hospitals and schools. Despite the benefits identified to date, analysis of the impact of ECHO on professional support networks and care integration has not occurred.

Recognising the challenges of fragmentation affecting the state-wide delivery of specialist paediatric care, CHQHHS introduced the ECHO model in 2017 [12, 13]. To improve child and youth health outcomes, a need for effective partnerships was identified across primary, secondary and tertiary healthcare and with non-medical institutions such as those within education, child safety, youth justice, and disability sectors. The ECHO model operationalises the 2018 CHQHHS Integrated Care Strategy [14] by providing a platform for bi-directional knowledge sharing between participants and panel members, in an environment that fosters interprofessional collegiality, empowerment of frontline providers, and communication between providers and services. The impacts of services and systems fragmentation compound the effects of other challenges to health equity, such as culturally inappropriate health services for Aboriginal and Torres Strait Islander people.

### Health and wellbeing of Aboriginal and Torres Strait Islander children and youth

A sense of cultural identity and nurturing family networks are among the resilience factors in the lives of many Aboriginal and Torres Strait Islander children and youth [15–17]. More than 6 in 10 (61%) Aboriginal and Torres Strait Islander people aged 10–24 recognised their traditional homelands or country, and over two-thirds (69%) were involved in cultural events [18]. Most young Indigenous people were connected to family and friends, describing as extremely or very important family relationships (74%) and friendships (67%) [18].

However, the effects of intergenerational trauma, racial discrimination, and socioeconomic disadvantage often disproportionately impact the health and wellbeing of Aboriginal and Torres Strait Islander young people. The child mortality rate for Aboriginal and Torres Strait Islander children was 141 per 100,000 in 2018 – more than twice the rate for non-Indigenous children comparatively (67 per 100,000) [19]. Potentially preventable hospitalisations are nearly twice as common in this cohort, at 21 per 1,000 compared to 11 per 100,000 for their non-Indigenous counterparts [18]. High to very high levels of psychological distress in the previous month have been reported by 33% of Aboriginal and Torres Strait Islander young people aged 15–24 years [20], associated with suicide and self-harm (13%), anxiety disorders (8%), alcohol use disorders (7%), and depressive disorders (7%) [20]. Currently, Aboriginal and Torres Strait Islander young people are over-represented in the youth justice system, at 26 times more likely to be in detention than their non-Indigenous counterparts and accounting for 56% (461 of 818) of young people in detention on an average night in the June 2022 quarter [21].

### Appropriate service provision and cultural brokerage

The holistic concept of health held by Aboriginal and Torres Strait Islander people considers not only the physical well-being of an individual, but refers to the “social, emotional and cultural well-being of the whole community in which each individual is able to achieve their full potential as a human being, thereby bringing about the total well-being of their community” [22]. Western medical models do not align with this definition and are therefore not appropriately designed to meet the needs of Aboriginal and Torres Strait Islander people. This can lead to challenges with health system engagement, such as feelings of intimidation due to unfamiliarity with the Western medical model, mistrust informed by current and/or historical experiences, varied experiences of safety in the physical environment, and interactions with staff not marked by openness, respect and culturally aware communication [23, 24].

A recent study found that one third of Aboriginal and Torres Strait Islander people who needed to see a health provider on at least one occasion in the previous 12 months were unable to attend the appointment [25]. Reasons provided included: high cost (34%) and wait times too long or service unavailable at the time required (33%).

A well-integrated Aboriginal and Torres Strait Islander workforce is crucial to the provision of health care for First Nations populations, and to the engagement of Aboriginal and Torres Strait Islander people in their own health [26, 27]. Aboriginal and Torres Strait Islander Health Worker and similar roles are multifaceted, with distinct but intersecting functions that include health promotion, clinical service provision, and cultural brokerage [28]. The concept of cultural brokerage encompasses both tangible and intangible approaches to health and wellbeing work, incorporating Aboriginal and Torres Strait Islander knowledges for the benefit of the client and service delivery systems [28, 29]. In this paper, the terms ‘cultural brokerage’ and ‘cultural broker’ encompass the unique mix of clinical and cultural skills that incorporate Aboriginal and Torres Strait Islander knowledges to promote the provision of more holistic care. Cultural knowledge nuanced in a clinical environment is an important enabler of the understanding, trust, and quality communication critical to culturally safe services [5, 28, 29]. Cultural brokerage is gaining traction in a variety of jurisdictions including North America, where there has also been historical disadvantage experienced by First Nations communities [30, 31].

To strengthen engagement of Aboriginal and Torres Strait Islander children and youth in their health care, strategies to support and sustain Aboriginal and Torres Strait Islander providers in the workforce are needed. Research indicates that Aboriginal and Torres Strait Islander Health Workers may improve attendance at appointments, acceptability of assessment and treatment recommendations, and enhance patient follow-up including referrals [32]. Roles that must be occupied by an Aboriginal and/or Torres Strait Islander person (identified roles) are under-represented in the health workforce. This may place unwelcome pressure on those working in these roles, and limit health services from improving responses to the needs within this population [25]. In 2019–2020, consultation with Aboriginal and Torres Strait Islander Health Workers in Queensland indicated several other concerns regarding these roles, including lack of awareness of role and scope, inconsistencies of practice, and a need for better access to peer and professional senior supports [33].

Following the establishment of a CHQHHS health equity strategy, the *Aboriginal and Torres Strait Islander Kids* ECHO network was launched in November 2021 with the objective of improving health system engagement with Aboriginal and Torres Strait Islander families through increased workforce capability for culturally responsive care. To provide culturally appropriate leadership and governance, a project steering committee was formed under the executive sponsorship of CHQHHS Executive Director of Aboriginal and Torres Strait Islander Engagement (co-author #5, a Kullali/Koa woman). Steering committee members were invited from state-wide leadership roles to provide advice to support the design, stakeholder engagement and implementation of an initial learning needs assessment, as well as initial promotion of the new ECHO network.

### Social network research

In this study, social network theories and methods are applied to provide an understanding of the connections occurring in an ECHO network. Social network research comprises a set of network concepts and theories as well as analytical methods used to systematically study relationships that exist between people, groups and organisations [34, 35]. In recent years, social network research has provided an innovative paradigm for health services research, contributing to a better understanding of contemporary health system problems including professional isolation, working in silos, barriers to adopting new practices, and team communication [36–38]. To date, there is one study that draws from network concepts to evaluate Project ECHO [39].

The overarching study aim is to investigate the knowledge sharing that occurs in the context of an ECHO network. Knowledge sharing can occur via formal relationships such as those arising between internal or inter-organisational colleagues with a shared objective (e.g. a health professional and their mentor), as well as via informal relationships that arise among colleagues who are also friends or family members. Informal relationships can also occur based on interactions in a workplace not necessarily involving a shared team or shared organisational objectives [40]. To capture knowledge sharing though formal and informal relationships the overall sharing of advice between colleagues was studied, as advice ties allow the circulation of ideas, problem-solving solutions and social support [41].

Formal and informal advice ties are equally important for sharing patient care knowledge and can be simultaneously present. The purpose of this investigation is to understand whether Project ECHO networks supported improved care integration via facilitation of advice exchange, in particular for Aboriginal and Torres Strait Islander children and young people. The following research questions are addressed:

1. What are the patterns of advice exchange within the ECHO network and across the broader network of providers?

Cross-organisation relationships can foster integration at a systems level, associated with integration between individual care providers and resulting in improved health and wellbeing outcomes. Boosting connectivity is an important mandate of CHQHHS. As such it is hypothesised that previous efforts in care integration will appear in network mapping.

*Hypothesis: Networks show well-connected areas, particularly between disciplines and sectors engaged in the ECHO network.*

2) What is the position of cultural brokers in the ECHO network as well as in the broader network of providers?

Cultural brokers incorporate Aboriginal and Torres Strait Islander knowledges into health care practices, for the benefit of the client and service delivery systems. Cultural brokers are a scarce knowledge resource in the health care environment in Australia.

*Hypothesis: Cultural brokers occupy strategic positions within the ECHO network and broader network.*

3) What are the most common relational tendencies that underpin knowledge exchange among professionals within the ECHO network as well as in the broader network of providers?

Social network analytical methods provide a way to systematically study the complex relationships that support the shared provision of care by individuals across discipline, service, sector and system boundaries. Note that the different relational tendencies studied will be outlined in the analytical method, below.

*Exploratory hypothesis: Identify the prevalent relational tendencies, providing guidance for future ECHO network interventions as well as broader CHQHHS care integration initiatives.*

This paper focuses on the implications of findings from the *Aboriginal and Torres Strait Islander Kids* ECHO network. A separate manuscript in development compares the results for both ECHO networks studied, and discusses in detail the multilevel analytical approach to healthcare social network research.

Studies have indicated that ECHO networks can create a CoP [42–47]. A CoP refers to a group with a shared interest in a real-life problem, who choose to interact regularly to learn from and with each other [3]. CHQHHS ECHO networks seek to align with the principles and values of a community of practice (CoP), therefore in this manuscript the terms ECHO network and ECHO CoP are used interchangeably.

## Methods

Seeking advice from a colleague was selected as the most appropriate relationship definition to capture the interactions that occur in interdisciplinary and cross-sector care, and to avoid the ambiguous nature of other relationship definitions such as communication frequency and collaboration [48]. A description of a relationship that involves advice-seeking was provided to respondents including examples for consideration of both formal and informal relationships that are of value in the provision of care to children and families.

Role and sector categorisation for all survey respondents and their nominees was completed by ECHO Network Coordinators (co-authors #1,4) based on the individual’s reported job title and organisation. This was done to facilitate analysis of relationships at both an interprofessional and cross-sector level. Individuals were assigned to one of the following categories for role: allied health, child safety, community elder, cultural brokerage, director/program lead, disability, education, medical, nursing, partnerships, project officer, youth worker, or other/unknown. For sector categorisation, individuals were assigned according to their organisation’s primary alignment with one of the following: primary health/community care, secondary/tertiary health, child safety/youth justice, disability, education, or other/unknown.

Individuals categorised as a cultural broker included the following identified and non-identified roles: Aboriginal Health Worker, Advanced Aboriginal Health Worker, Aboriginal Mental Health Worker, Cultural Officer, Indigenous Health Worker, Cultural Support Worker, Indigenous Liaison Officer, Indigenous Health Liaison Officer, and Indigenous Education Liaison Officer. Cultural brokerage is sometimes expected of Aboriginal and Torres Strait Islander staff members regardless of whether this is included in their role description, often without remuneration. Therefore, professionals occupying medical, nursing, allied health and other roles who identify as Aboriginal and/or Torres Strait Islander and may incorporate cultural and cultural knowledge in their delivery of holistic care were not categorised as a cultural broker.

### Research team

The first two authors served as principal investigators for the study, forming a health services research partnership between CHQHHS and the Institute for Social Sciences Research at The University of Queensland. Co-authors from the Project ECHO Hub at CHQHHS (co-authors #3,4 and 6) contributed expertise implementing and evaluating ECHO CoPs addressing child and youth health priorities. Co-author #5 identifies as Aboriginal and/or Torres Strait Islander. Through their dual role as project steering committee lead and facilitator of the *Aboriginal and Torres Strait Islander Kids* ECHO CoP, they contributed executive governance and sponsorship, cultural insight and advice, as well as recommendations for stakeholder engagement.

### Data collection and participant engagement

The network boundary included professionals residing in the state of Queensland who registered as ECHO participants, as well as ECHO panel members. ECHO CoP members were eligible for the study if they attended at least one session during the survey development period (January-May 2022). Network data were collected in two ECHO CoP: network 1 (*Aboriginal and Torres Strait Islander Kids* ECHO), and network 2 (*Navigating Paediatric Disability* ECHO*)*. The latter has been operational since 2020, and was selected for comparison purposes. During a routine ECHO session participants were invited to voluntarily take part in the study. Study information was provided to all participants by email, with link to the Qualtrics survey (see Additional file 1).

**Fig. 1:**
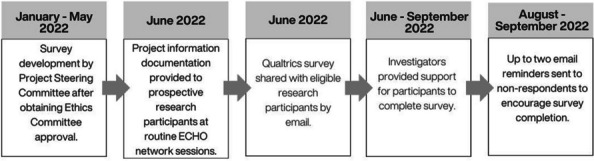
Data collection process flow chart

Network 1 participants were from varied geographic locations in Queensland. Participants occupied diverse roles including medical, nursing, allied health, education and social service roles. Organisational settings included mainstream health, education, child safety and disability organisations as well as non-government, community controlled and private organisations. ECHO network participants were asked to nominate the people they seek advice from both within and outside the ECHO group. They were also asked to indicate the potential for information exchange between their external nominees. For further information regarding the data collection process, see Fig. 1.

### Analytical method

Social network research is the investigation of social structures using network and graph theory, and for this study examined formal and informal advice-seeking relationships. In social network visualisations, a ‘node’ refers to an individual within a network. Nodes are connected by ties, representing the relationships between them. In this study ties are considered undirected, since the connection through which knowledge and advice is obtained opens the possibility for reciprocity. Patterns of advice tie formation tend to be highly generalisable. Relational tendencies such as density, transitivity, homophily and activity can explain why ties arise in almost all advice exchange networks. In the context of professional roles, the baseline tendency to seek advice (*density*) tends not to be reciprocal, mainly due to hierarchical differences [49]. *Transitivity* measures the tendency for an individual to seek advice from those who provide advice to someone’s advisor, creating a triad pattern of connectivity. *Transitivity* tends to reinforce the presence of hierarchical dynamics [50] and shared advisors [51]. Another important mechanism, *activity*, is the tendency to actively reach out for advice. The more there are active nodes, the more the network can be centralised suggesting the presence of consultative mentors and leaders. Studies have demonstrated that in work-related advice networks, some people are generally more trusted than others and more frequently act as advisors [52]. *Homophily*, based on individual attributes rather than ties, is the tendency to seek advice from those with a shared attribute. *Homophily* indicates the presence of bridges that incentivise information flow among individuals with similar types of knowledge and background, due to working in the same sector or having the same role. The presence (or absence) of these relational tendencies can be used to examine, in each network, the overall level of segregation, clustering, integration and connectivity, as well as governance, efficiency, and trust.

**Fig. 2:**
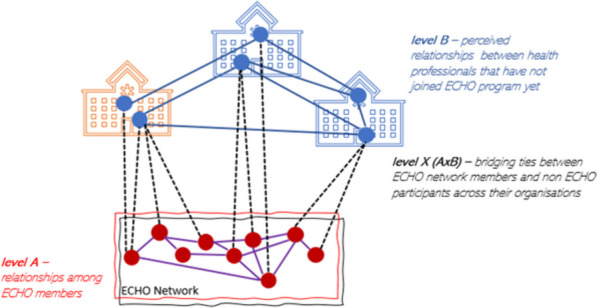
Schematic view of the multilevel advice-seeking relationships among ECHO CoP members and their nominated advisors

Statistical models called Exponential Random Graph Models (ERGMs) allow examination of relational tendencies that are not possible to analyse with conventional statistical methods. Using the extension of these models for multilevel networks, it is possible to investigate how relational processes occur within and across overlapping networks, and how these relate to tie formation while considering interdependency of network observations on multiple levels [53, 54]. A Multilevel Exponential Random Graph Model (MERGM) was completed separately for each ECHO CoP. Each network considers respondents and nominees at different levels. Figure 2 depicts a schematic visual representation of the three level networks – A, B and X – for each ECHO CoP.

Network 1 comprised *N* = 44 individuals at level A, including ECHO CoP members and their nominees who were also ECHO CoP members. At level B there were *N* = 179 individuals, these were the advisors nominated outside ECHO. There were three different sets of undirected ties: at level A the advice-seeking ties within ECHO CoP members, at level B the ties among external nominees and at level X the cross-level bridging relationships between ECHO CoP members and their nominated individuals. Network 1 comprised *N* = 96 ties at level A, *N* = 229 ties at level B, and *N* = 239 across levels (level X).

MERGMs consider how ties occurring at level A are interrelated with those occurring at level B, recognising that some relational tendencies are occurring uniquely within each level, and some across levels. In addition to the study of relational tendencies based on shared role and sector (density, transitivity, activity and homophily), several other network mechanisms are of interest. In particular, the likelihood of seeking advice from an advisor’s advisor, creating small clusters and three-person subsystems (i.e., triangles) of advice exchange. Moreover, ECHO CoP member advice-seeking patterns with those outside the CoP with similar patterns of connections, as well as whether professionals tend to assume a brokerage position by actively linking professionals within and outside ECHO (i.e., across levels A and B).

## Results

### Sample characteristics

The survey was completed by 37 out of 53 (69.81%) eligible participants from network 1, and 31 out of 66 (46.96%) eligible participants from network 2. On average, respondents participated in 2.03 different ECHO networks. There were 8 respondents who participated in both ECHO networks. The following results and discussion are relevant to the findings from network 1.

The sample of 37 network 1 participants included 12 (32.43%) Aboriginal and/or Torres Strait Islander people. Respondents predominantly supported children and/or youth in a major region (70.27%), remote (21.62%) or rural (8.11%) area.

### Research question 1 – Advice exchange patterns

**Fig. 3:**
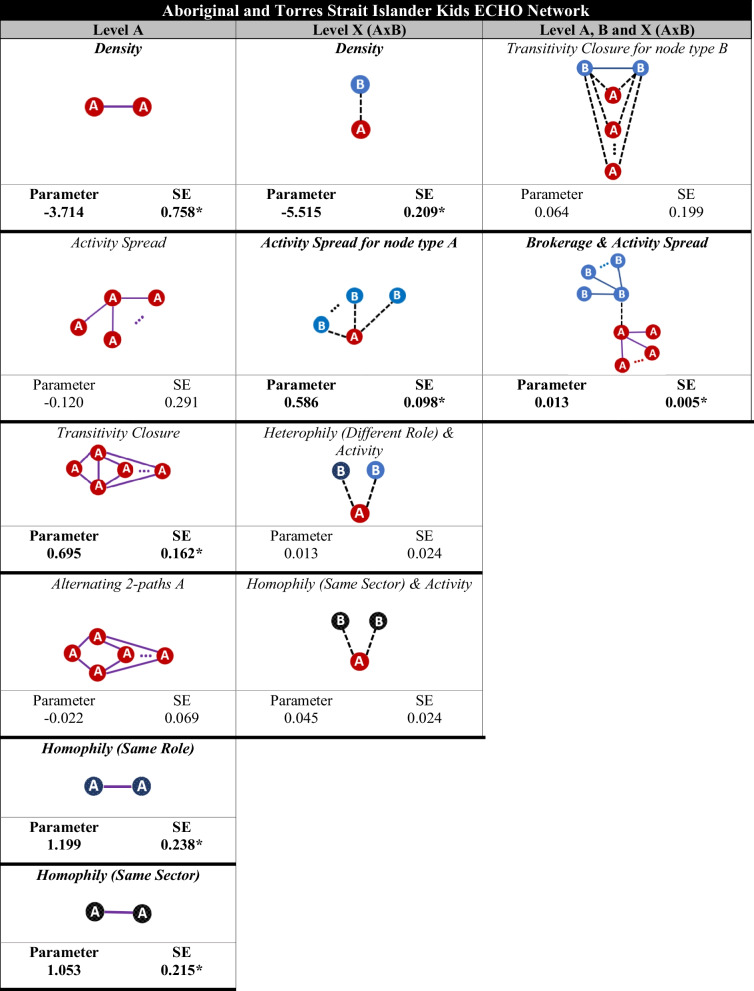
MERGM results and visualisations for advice exchange relational tendencies. Parameters with an asterisk (*) are significant, indicating that they are either more commonly (positive parameters) or less commonly (negative parameters) observed than that would have been found by chance

Among ECHO CoP members studied a positive and significant tendency for transitivity was noted, that is, the presence of small clusters of advice ties (Fig. 3, transitivity closure parameter, level A). The analysis of dyads, or pairs of individuals who have a direct relationship, indicated that knowledge-sharing may be incentivised by shared role and sector. There was a tendency for ECHO CoP members studied to exchange advice with co-members of the same role (homophily [same role], level A). Similarly, advice exchange ties within the CoP were more likely to occur between co-members from the same sector (homophily [same sector], level A). The tendency to form advice exchange ties with those outside the ECHO network was non-significant for role (heterophily [different role] & activity, level X) and sector (homophily [same sector] & activity, level X).

Diffuse organisational links were present, based on the advice-seeking relationships that exist between ECHO CoP members and their nominees. The strength of organisational links was determined based on the number of advice-seeking ties between individuals affiliated with any two organisations. Individuals were affiliated with 28 organisations from health, education and community-controlled sectors, and from 20 postcodes across Queensland. Relational ties supported by the ECHO CoP serve as advice-exchange links between 7 Hospital and Health Services, 10 Aboriginal and Torres Strait Islander Community Controlled Health Organisations, 3 Department of Education regions, 2 Primary Health Networks, and several other advisory or frontline organisations.

### Research question 2 – Position of cultural brokers

**Fig. 4:**
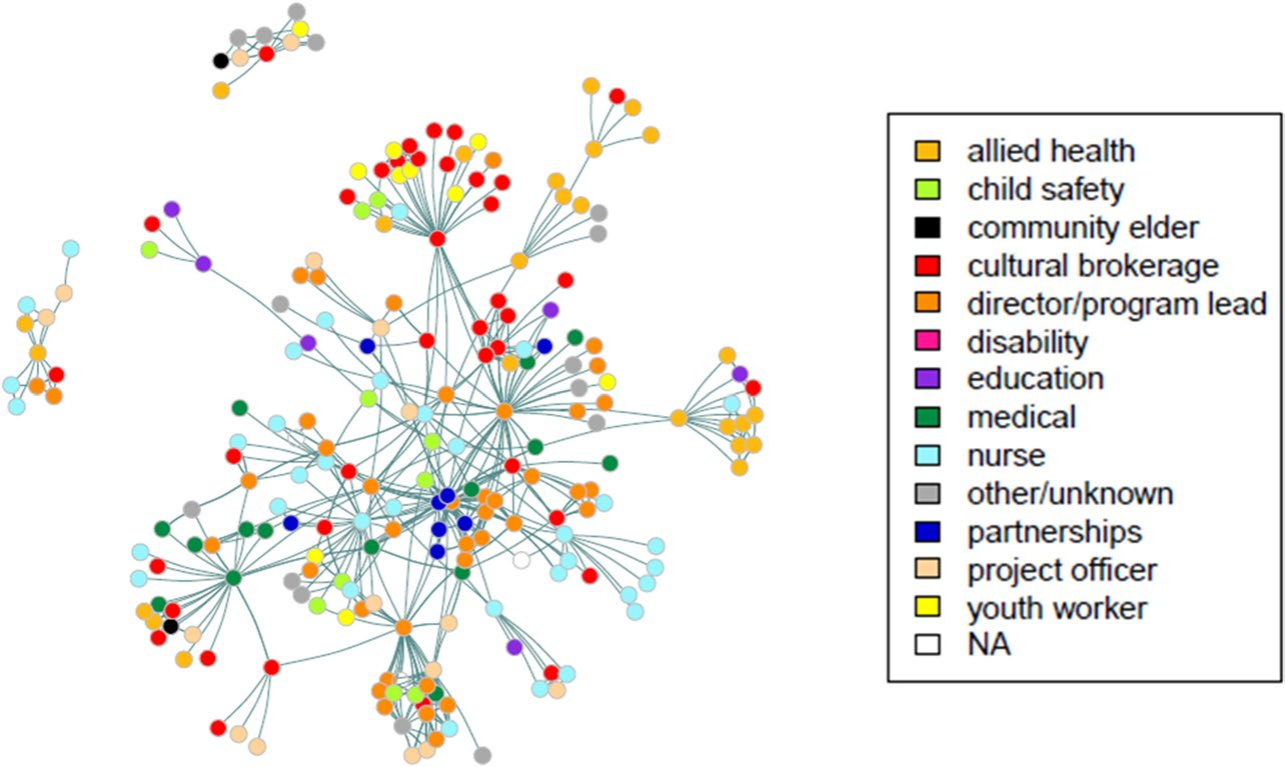
Advice-seeking relationships among ECHO CoP members and their nominees; nodes categorised by role

**Fig. 5:**
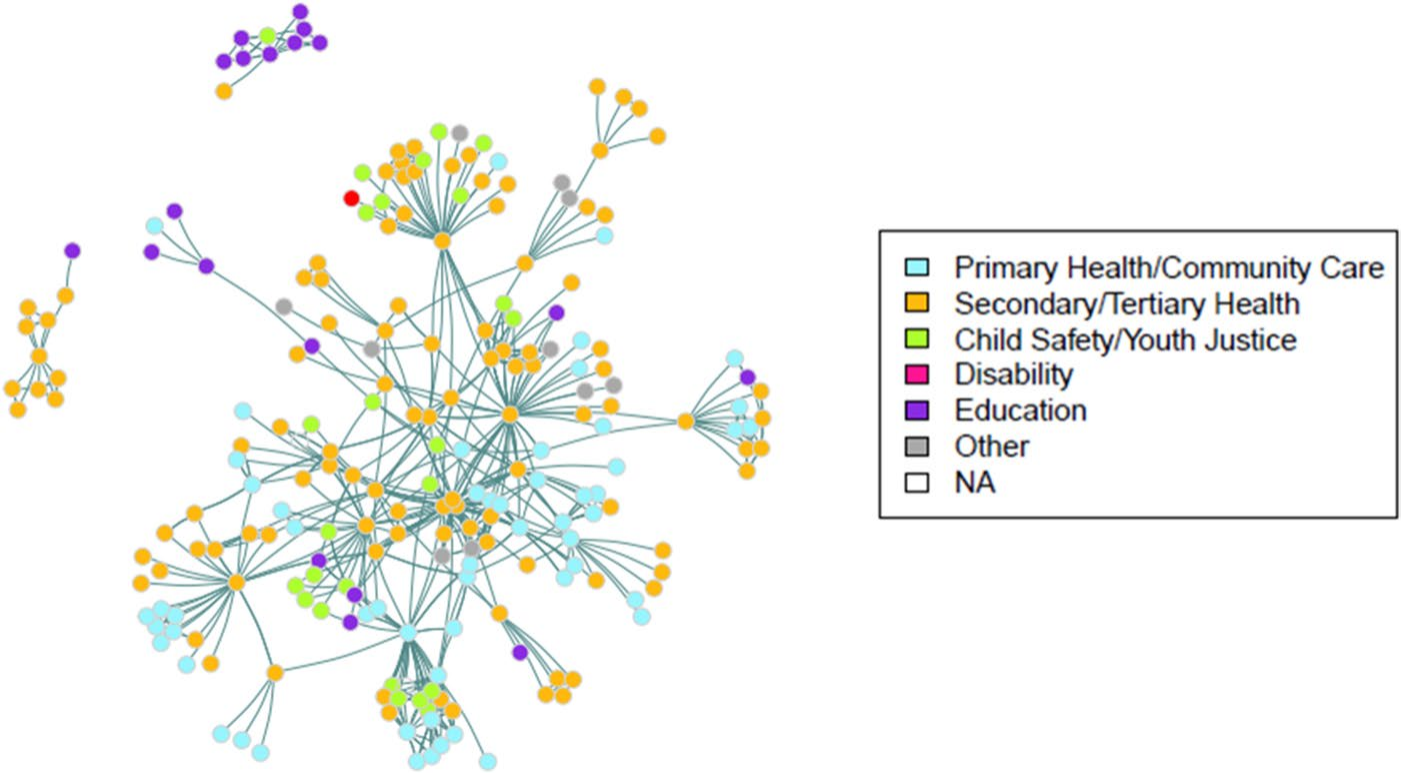
Advice-seeking relationships among ECHO CoP members and their nominees; nodes categorised by sector

The advice exchange connections between individuals were displayed in network maps (Figs. 4 and 5). Individuals were represented by nodes or dots, and undirected advice exchange ties represented by the lines between them. Nodes were categorised by role (Fig. 4), and by sector (Fig. 5).

The 223 individuals in network 1 (levels A and B) included 38 individuals with cultural brokerage as a primary function of their role, working within the secondary/tertiary health sector (9% of total cohort), primary health/community care (5%), child safety/youth justice (3%), and education (1%).

Mapping by sector demonstrates the broader community within which the cultural brokers fulfil their role, comprising individuals from secondary/tertiary health sector (52%), primary health/community care (23%), child safety/youth justice (12%), education (8%) and other/unknown (5%).

### Research question 3 – Exploratory analysis of relational mechanisms

ECHO CoP members studied held advice exchange ties with multiple colleagues beyond the ECHO network (Fig. 3, activity spread for node type A, level AxB). MERGM analysis indicated that the presence of brokerage tendencies across levels was positive and significant (Fig. 3, brokerage and activity spread). Level B colleagues were intermediaries for the flow of information from ECHO network sessions to the broader community of providers. In social network research, the term brokerage refers to the position of an individual that connects two nodes that are otherwise unlinked [55]. Brokers occupy “structural holes” between nodes or clusters [55]. Being a broker means being in a privileged position to pass on information and, with it, to reduce network fragmentation. In this sample, those who occupied a brokerage position were also those with a high number of advice exchange relationships (Fig. 3, brokerage and activity spread, level A, B and X). Specifically, those who occupied a brokering position outside the ECHO CoP (across levels A and B) tended to also be more connected with other professionals within the CoP.

Although the MERGM analysis did not reveal brokerage tendencies unique to cultural brokers, this may be attributed to the limited number of individuals fulfilling the role or possibly due to challenges parameterising this particular tendency.

## Discussion

This study examined the formal and informal advice-exchange relationships occurring in the context of the *Aboriginal and Torres Strait Islander Kids* ECHO CoP and allied providers. Though the findings pertain to the specific ECHO CoP studied, they are of relevance to ECHO CoP and healthcare teams globally that seek to integrate clinical and cultural knowledge for more holistic patient care. The manuscript focuses on advice exchange patterns, cultural brokers, and dual activity/brokerage relational tendencies. Since the research literature discussed in this section includes a mix of domestic and international studies and no studies that have been Aboriginal and Torres Strait Islander led, relevance of literature to the Aboriginal and Torres Strait Islander context will need to be considered carefully.

Brokers, both those who hold general knowledge sharing brokerage positions as well as those in roles dedicated to cultural brokerage, are well represented in this sample. Unique functions of brokers include facilitating flow of information and resources, supporting knowledge exchange, and coordinating contributions across the network [56]. Moreover, brokers who connect individuals across disparate groups *(boundary spanners)* are of value to a network in their ability to engage with perspectives outside their own profession, culture and mindset, contributing to the development of trust and reciprocity [57, 58].

The findings align with recent literature recognising the value of distributed brokerage in cross-level networks, for the delivery of integrated services [59, 60]. The unique role played by those who bridge a gap between individuals and are themselves connected to many, referred to by Fujimoto and colleagues [60] as *brokerage-centrality conjugates*, contributes to collaborations at an inter-organisational level. The distributed brokerage-centrality conjugates in two functionally distinct groups (level A and B networks) have a privileged position in improving knowledge exchange and coordination supporting professional engagement across traditional boundaries for the delivery of coordinated, collaborative and person-centred care.

The function of knowledge brokers in the context of ECHO CoP has not been studied in-depth. One study from an HIV clinical care ECHO included staff who did not participate in ECHO but were colleagues with a provider who did [61]. A statistically significant improvement in viral load suppression was demonstrated in patients treated by a provider co-located with an ECHO participant, compared to the control group of providers not participating and not co-located with an ECHO participant [61]. The present study builds on these findings, recognising the value of participants who become brokers and are well-placed to facilitate diffusion of knowledge and resources within their local environment. The increase in connectivity afforded by brokers facilitates the coordination and collaboration necessary for effective care integration.

The experience of burden due to others’ over-reliance on them may be a risk to the broker [56]. In the case of cultural brokers, the multiple roles and responsibilities held towards their communities as well as their workplaces can sometimes overlap causing an additional challenge [5, 62–65].

Knowledge afforded by cultural brokers can contribute significant value in the development of collaborative solutions addressing gaps in service delivery and care. All professionals have a responsibility to make necessary adjustments to routine care to best meet cultural needs, regardless of whether their role requires specific or incidental cultural service provision. One-to-many knowledge sharing relationships such as those that exist in an ECHO CoP offer an opportunity to support professionals to make these adjustments, enabling providers from diverse disciplines, sectors, and locations to engage in a culturally nuanced way with health consumers. The prioritisation of psychological safety in an ECHO CoP permits open-handed sharing of rich contextual and experiential knowledge.

In view of the unique contribution of cultural brokers to enhanced service delivery as well as the challenges they face, CoPs offer a sustained peer support mechanism contributing to sense of belonging, increased confidence in problem-solving, and strengthened sense of professional identity [3]. Mentorship and mutual support networks have already been advocated for as necessary supports for cultural brokers in the literature [32, 62, 64, 66, 67].

### Network intervention

To increase awareness and nurture the connections and collaboration occurring between ECHO CoP members and their colleagues, a network intervention was completed in the form of a digital booklet disseminated to CoP members and other stakeholders (see Additional file 2). Artwork inspired by the relationships occurring in an ECHO CoP was created by a contemporary Aboriginal artist and incorporated into the design of the booklet. The booklet highlighted the value of connections in everyday work objectives and based on common trends, identified strategies to foster integration between providers, services and systems. Two key recommendations were provided: 1) share knowledge gained at ECHO with like-disciplines colleagues or invite them to join an upcoming session, and 2) schedule a regular day to become a knowledge broker by connecting colleagues who don't know each other and exploring opportunities for their collaboration. Since ECHO networks are learner driven, they continuously evolve in response to participant engagement and the learning agenda. Recognising the importance of relationships and the role of brokers for knowledge sharing contributes to the future reach and influence of the CoP.

### Limitations and future directions

Engagement with study respondents in a state-wide virtual CoP was challenging. Although twice-weekly virtual drop-in sessions were offered to assist with survey completion, uptake of these was low. It is recommended that future social network studies investigating virtual CoP prioritise planning for stakeholder engagement, employing follow-up aids to survey completion and face-to-face interaction wherever possible. Efforts were made to explain informal and formal advice-seeking relationships, however the possibility that study respondents did not have a common understanding of these terms cannot be excluded. Privacy and confidentiality are a particular sensitivity for professionals included in this sample, and one participant reported discomfort providing the name of role of colleagues without their permission. Enhanced engagement in virtual drop-in and follow-up sessions would help to mitigate the impact of these concerns on survey completion. The recruitment of pre-registered ECHO participants to the study presents a source of sample and volunteer bias, with respondents more likely to understand the value of interdisciplinary and cross-sector connection. Lastly, respondent report of those they seek advice from was dependent on recall, and some contacts may have been missed.

Since there was insufficient data to study the relational tendencies of cultural brokers, additional research is indicated to further describe this cohort. Cultural brokers’ perceptions of the impact of ECHO CoP on their work, and co-participant integration of cultural care practices are additional questions for further research.

## Conclusion

Social network theories and methods revealed the advice exchange patterns present in an ECHO CoP and across the broader network of providers linked to members of the CoP. Brokers who bridge the gap between the CoP and the broader network of providers are positioned to advance the knowledge diffusion objectives inherent to any ECHO CoP. The findings of this study highlight the connectivity afforded by brokers, enabling the coordination and collaboration necessary for effective care integration. Inclusion of cultural brokers in an ECHO CoP provides them with peer group support and mentorship, while concurrently cultivating relationships that facilitate diffuse integration of cultural and clinical knowledge.

### Supplementary Information

**Additional file 1.** ECHO Relationships Project Survey Instrument.

**Additional file 2.** ECHO Relationships Project Booklet.

## Data Availability

The datasets analysed during the current study are available from the corresponding author on reasonable request.

## Abbreviations

ATSICCHO: Aboriginal and Torres Strait Islander Community Controlled Health Organisation
CHQHHS: Children’s Health Queensland Hospital and Health Service
CoP: Community of Practice
ECHO: Extension for Community Healthcare Outcomes
ERGM: Exponential Random Graph Model
MERGM: Multilevel Exponential Random Graph Model
