# Supplementary material for: Healthcare social network research and the ECHO model™: Exploring a community of practice to support cultural brokers and transfer cultural knowledge
Source: BMC Health Serv Res. 2024 May 1;24:558. doi: 10.1186/s12913-024-11024-w (PMC11062014; doi:10.1186/s12913-024-11024-w)
Supplement: Supplementary file 1 — Additional file 1. ECHO Relationships Project Survey Instrument. [file 12913_2024_11024_MOESM1_ESM.pdf]

ECHO Relationships Project

Welcome **Thank you for participating in this project!**

Children's Health Queensland and the University of Queensland pay our collective respects to the Traditional Custodians of the land on which we walk, talk, work and live. We acknowledge and pay our respects to Aboriginal and Torres Strait Islander Elders past, present and future.

We are interested in knowing more about who you network with to support Aboriginal and Torres Strait Islander peoples' health.

This will bring awareness on how connections are shaped and will help us identify gaps and barriers that we can change together.

[Video message from Network Facilitator]

[Participant Information Sheet and Consent Form]

Part 1 – Participant Information

1 Please, provide your first and last name:

____

2 My preferred pronouns are:

- ◯ He/Him/His (5)
- ◯ She/Her/Hers (6)
- ◯ They/Them/Theirs (4)
- ◯ Prefer not to answer (7)

3 Are you of Aboriginal or Torres Strait Islander origin?

- ◯ No (4)
- ◯ Yes, Aboriginal (5)
- ◯ Yes, Torres Strait Islander (6)
- ◯ Yes, both Aboriginal and Torres Strait Islander (7)

Display This Question:

If Are you of Aboriginal or Torres Strait Islander origin? = Yes, Aboriginal

4 Who's your mob? Where's your country?

____

Display This Question:

If Are you of Aboriginal or Torres Strait Islander origin? = Yes, Torres Strait Islander

Q581 Who's your mob? Where's your country?

____

Display This Question:

If Are you of Aboriginal or Torres Strait Islander origin? = Yes, both Aboriginal and Torres Strait Islander

Q580 Who's your mob? Where's your country?

____

5 What sector do you work in?

- ▢ Health (1)
- ▢ Education (2)
- ▢ Disability (3)
- ▢ Child Safety (4)
- ▢ Human Services (5)

6 In your work, where do the majority of people you support live?

- ▢ City (1)
- ▢ Major region (2)
- ▢ Rural (3)
- ▢ Remote (4)

7 What organisation do you work for, and what's your role there (e.g. Children's Health Queensland, Aboriginal Health Worker etc.)?

____

8 What knowledge and strengths do you bring to the ECHO network? (Tick all that apply)

- ☐ Cultural (4)
- ☐ Clinical (5)
- ☐ Social and Welfare (6)
- ☐ Justice (7)
- ☐ Foster care (8)
- ☐ Education (9)
- ☐ Disability (10)
- ☐ Advocacy (11)
- ☐ Policy and project (12)
- ☐ Other (13)

- Do you currently attend any other Children's Health Queensland ECHO networks?

- ○ Yes (1)
- ○ No (2)

Display This Question:

If Do you currently attend any other Children's Health Queensland ECHO networks? = Yes

- Which one?

☐ Aboriginal and Torres Strait Islander Kids Health and Wellbeing (4)

☐ Adolescent Health and Wellbeing (5)

☐ Child Protection: Responding to Vulnerable Children and Families (6)

☐ Children, Adolescents and Young Adults with Complex Pain (7)

☐ Navigating Paediatric Disability (8)

☐ Paediatric Gender Health Care (9)

☐ Paediatric Palliative Care (10)

☐ Supporting Teams Caring for Type 1 Diabetes (11)

Part 2 – Social Networks

2 Please select all the people **inside the ECHO Network** with whom you tend to reach out and connect. For example, for general advice and guidance, for cultural advice, to share ideas...

This is the list of people you selected. Please, click next to continue.

You may have this type of relationship with people **outside the ECHO network** as well, who work at your organisation or at the organisations of other ECHO participants.

Please select the organisations that apply from the list below.

4-1 At ***[organisation name]***, who do you tend to reach out and connect with? (e.g., for general advice and guidance, for cultural advice, to share ideas..)

	Please write their name and role in the spaces provided below. If you do not have 6 people to nominate, it's OK.	
	Name, Surname (1)	Role, if known (2)

For each person nominated inside and outside the ECHO network, please respond to the following:

1 How close are you to ${lm://Field/1}?

- ◯ not much (1)
- ◯ quite (2)
- ◯ a lot (4)

---

2 Is ${lm://Field/1} a member of your family?

- ◯ no (1)
- ◯ yes (2)

Part 3 – Alter-Alter Ties

How likely is it that all the people you have nominated also know each other, and share knowledge and advice?

For each person in the box, **drag and release** the name/s of people you think are connected with that person as well.
