# Supplementary material for: Healthcare social network research and the ECHO model™: Exploring a community of practice to support cultural brokers and transfer cultural knowledge
Source: BMC Health Serv Res. 2024 May 1;24:558. doi: 10.1186/s12913-024-11024-w (PMC11062014; doi:10.1186/s12913-024-11024-w)
Supplement: Supplementary file 2 — Additional file 2. ECHO Relationships Project Booklet. [file 12913_2024_11024_MOESM2_ESM.pdf]

Children's Health Queensland

ECHO Relationships Project

Our connections are an important factor in what we know, how we think and what we do. They help us to learn, and to thrive. Project ECHO® networks are online communities of practice that create opportunities for people to connect.

What can you do to nurture connections, and bridge the gap between providers, services, and systems?

Developed in collaboration with

The University of Queensland Australia

Project ECHO®

Queensland Government

ECHO Network study

At Children's Health Queensland's Project ECHO® Hub we aim to support the provision of coordinated care for children and their families. Project ECHO is a knowledge sharing model connecting people through regular videoconferences. These online communities bring together like-minded people to share ideas, collaboratively problem solve challenges, and build trust between systems and sectors.

A research project by Children's Health Queensland and the University of Queensland has applied Social Network Research and Analysis to map out the professional and personal connections of participants in online ECHO networks. The project was designed to help us understand the connections occurring in an ECHO network, and how group members can get the most out of their participation in the network.

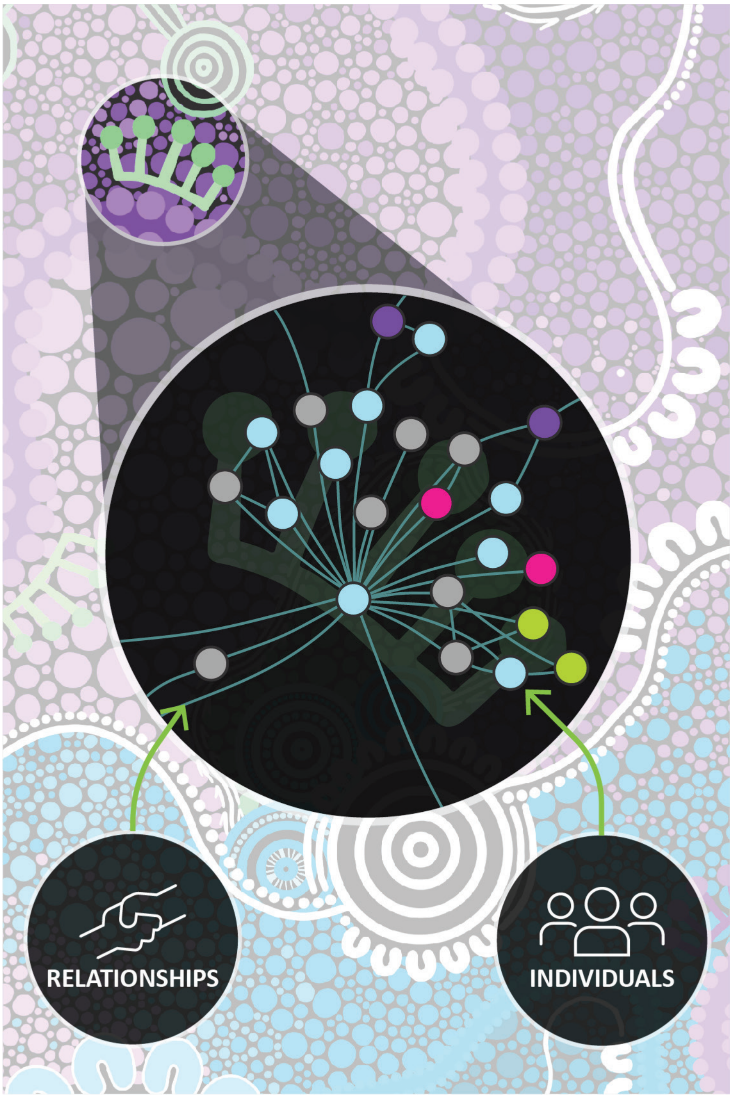

Social Network Research and Analysis provides the analytical and theoretical tools for systematically analysing a network. In this case, we applied some Social Network Research and Analysis tools to analyse the formation of relationships within ECHO networks. In traditional network visualisations, individuals are represented as dots (circles) and relationships among them as ties (lines).

What we did

New and strengthened connections in each ECHO network enable people to link in more effectively with other care providers, services, and sources of knowledge, to deliver the best care for our families.

We studied two ECHO networks, each with a distinct objective:

1. **Aboriginal and Torres Strait Islander Kids ECHO:** to connect stakeholders to problem solve challenges in the provision of culturally appropriate care for First Nations families.
2. **Navigating Paediatric Disability ECHO:** to build a cross-sector community supporting children and young people living with a disability.

We asked participants who attended at least one session of the ECHO network in January to May 2022 to identify those they connect with to seek general, cultural or other type of advice, both inside and outside the ECHO network.

ECHO Network maps

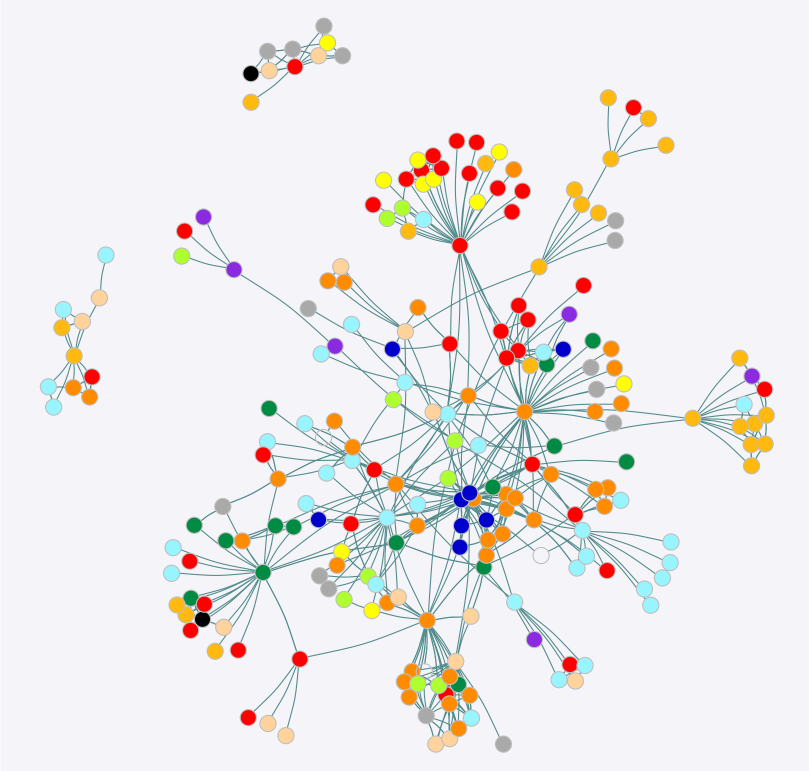

Aboriginal and Torres Strait Islander Child and Youth Project ECHO group

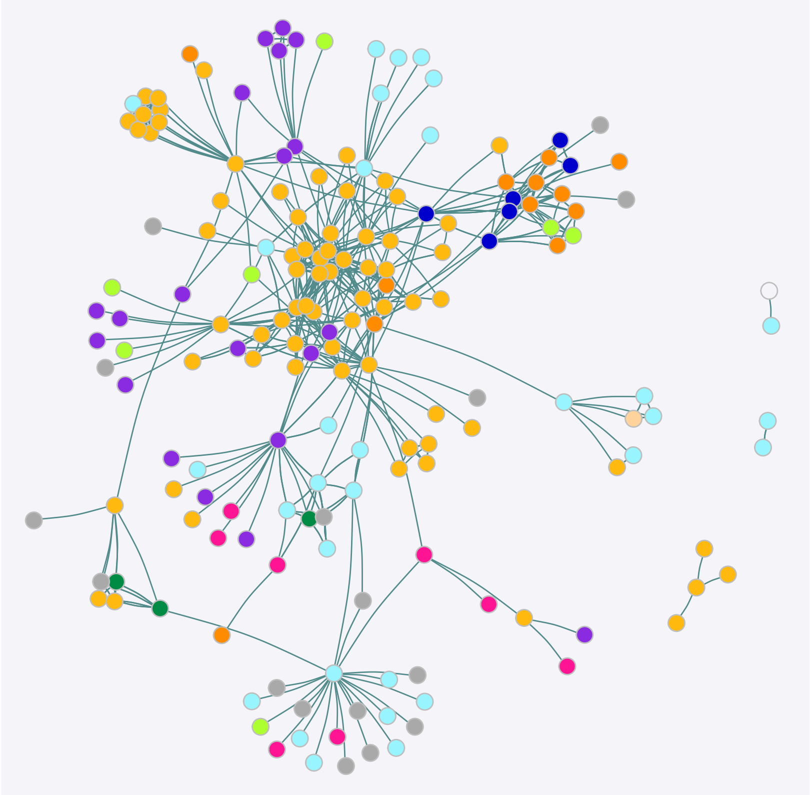

Paediatric Disability Project ECHO group

Dots represent professionals, and ties between them are relationships in which advice-seeking occurs.

Roles

- allied health
- child safety
- community elder
- cultural brokerage
- director/program lead
- disability
- education
- medical
- nurse
- other/unknown
- partnerships
- project officer
- youth worker
- NA

Findings

We found that:

- Individuals studied in our sample were less likely to share advice with colleagues of the same discipline.
- ECHO networks provide clustering opportunities that are distributed in several areas of the networks. Connections link participants from different institutions. This promotes knowledge sharing among like-minded professionals in different settings.

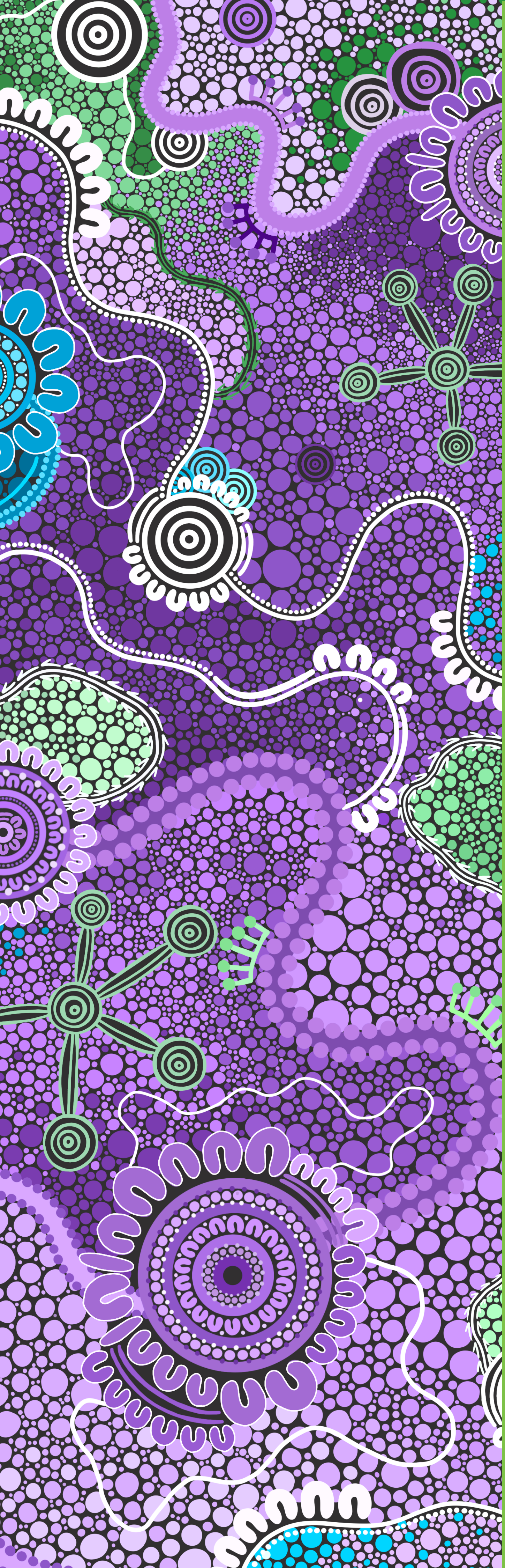

Here are two things you could do to improve your working relationships

1. Invite your like-discipline colleagues inside or outside your institution to join ECHO Networks and share what you're learning with them. This creates the opportunity for them to learn from you and to share knowledge and information with their own network of colleagues, family and friends.
2. Dedicate a day on a regular basis to network and to become a knowledge broker. Invite a couple of colleagues who may not know each other for coffee. You can use this opportunity to share how important their individual skills and knowledge are to you, and why you think they might benefit from collaborating and sharing knowledge with each other as well. This increases connectivity at a broader level.

Curious to find out more about the science behind this research? Get in touch!

E: ECHO.CHQ@health.qld.gov.au
W: http://echo.qld.gov.au

Acknowledgements

Children's Health Queensland's Project ECHO® and The University of Queensland acknowledge the Traditional Owners of the land and pay respect to Elders past, present and future.

Original cover artwork by Maggie-Jean Douglas (Gubbi Gubbi). Graphic design and research translation by Gina Nuttall at The Institute for Social Science Research, The University of Queensland.
